# Compassion—A key to innovation: What promotes and what prevents innovation in organizations?

**DOI:** 10.3389/fpsyg.2023.1058544

**Published:** 2023-02-27

**Authors:** Jenni Spännäri, Elina Juntunen, Anne Birgitta Pessi, Pirjo Ståhle

**Affiliations:** ^1^Faculty of Theology, University of Helsinki, Helsinki, Finland; ^2^School of Engineering, Aalto University, Helsinki, Finland

**Keywords:** compassion, innovation, organization, workplace, organizational culture, qualitative study

## Abstract

Innovation is crucial for the survival and wellbeing of organizations in volatile, rapidly changing societies. However, the role of profound human capability, compassion, and innovation has not been adequately investigated. This article sets out to explore the factors preventing and promoting innovation in organizations, asking how compassion is connected to these factors, and how compassion could boost innovation. We approach innovation as a complicated multilevel phenomenon, emerging from interactions between individuals and the work context. Our view of compassion includes both compassion and copassion—responding both to the suffering and joy of others. Our material was collected from nine focus group interviews, organized in Finland in 2017, in private, public, and third-sector organizations. The material was analyzed by two researchers, using an adapted grounded theory methodology. We found four core factors capable of either promoting or preventing innovation: (1) the strategy and structures of the organization, (2) resources, especially time, (3) working culture; and (4) the dynamics of interaction between individuals and the community. Our key conclusion, fruitful to theorizing both innovation and compassion, is that for innovation to flourish, compassion is to be cultivated throughout an organization. It is not a single variable or practice, and it is in many ways in a key position regarding innovation: the existence of it promotes innovation, but the lack of it prevents innovation. Thus, organizations aiming for innovation should seek multifaceted understanding and skills in compassion.

## 1. Introduction

Innovation is crucial for the survival and wellbeing of organizations in volatile, rapidly changing societies (see Bagheri et al., 2019; Mumtaz and Parahoo, 2020; Sharma et al., 2022). Innovation is also a human trait, built into and during the process of evolution (Reader et al., 2016). But how to recognize, access, and foster successful innovation and innovativeness? How to encourage new ideas for the greater good and for a longer time span, and not only for short-term profit? In particular, what is the role of compassion, which is a deeply rooted human trait, considered by Darwin to be the strongest force in evolution? (Darwin, 2004; Ekman, 2010).

Many organizations are being confronted by the challenges of finding the innovatory potential of their employees. Technical and structural elements, as well as the social aspects of the work environment, might influence employees’ capacity for innovation (Mumtaz and Parahoo, 2020). Still, we have a very limited understanding of the social, emotional, and motivational factors fostering employees’ innovativeness at work (Bammens, 2015).

In this study, we approach innovation as a multilevel phenomenon (see also Mumtaz and Parahoo, 2020; Sharma et al., 2022). Employees’ innovativeness is a complex process in which innovativeness can be seen to occur as a result of several interconnected factors (Parzefall et al., 2008). In this study, we are interested in how individual-level and contextual factors, and their synergy, affect employees’ innovation in the work context. We analyze factors at the individual, team, and organizational levels. In particular, we articulate why and in what ways compassion may enable innovative behaviors. How might compassion support and foster innovative efforts in organizations?

The key research questions in this article are as follows:

What are the factors preventing and promoting innovation in organizations?

How is compassion connected to these factors?

How could compassion boost innovation?

Compassion at work and within organizations has been researched as being instrumental in, for instance, coaching, *ad hoc* organizing, prosocial behavior during challenging times, and other processes central to developing and transforming organizations. Compassion has numerous proven benefits for organizations. For example, it has been shown to bring about an untapped organizational capability, contribute to fostering a climate of forgiveness, and facilitate the development of social entrepreneurship (Avramchuk et al., 2013). Miller et al. (2012) have looked at the development of compassion and social reform and entrepreneurship. The research shows that compassion plays an important role in identifying people’s suffering, needs, and expectations, as well as in developing new social practices. According to Miller et al., compassion, above all, arouses and strengthens prosocial motivation, which leads to flexible thinking and commitment to action. All this is crucial to innovation too.

## 2. Innovation and compassion

### 2.1. Innovation and innovativeness

Innovation and innovativeness have been the focus of research interest for approximately 30 years. Research has looked at innovation from the perspectives of leadership and management styles, work environment factors, organizations, teams, networks, and individuals (Scott and Bruce, 1994; King and Anderson, 2002; Hunter et al., 2007; de Jong and Den Hartog, 2010). In this article, we use innovation and innovativeness to describe the vast field of both innovative attitudes and behavior. Thus, we define innovation as both creativity, such as the generation of new ideas and implementation, such as applying new knowledge or improving processes (see de Jong and Den Hartog, 2010).

Innovative work in the past has often been misunderstood. It has easily been considered only in terms of the development of creative ideas. Creativity is a discernible part of innovative work, but it is also simply one part of innovativeness (Martins and Terblanche, 2003; de Jong and Den Hartog, 2010). Employee innovativeness goes beyond creativity to include the adoption, production, and implementation of novel and useful ideas (Scott and Bruce, 1994; Mumtaz and Parahoo, 2020). Innovativeness at work includes actions such as seeking out new ideas, championing ideas at work, and securing funds/planning for the implementation of ideas (Scott and Bruce, 1994). This type of behavior requires risk-taking and out-of-the-box thinking, and perhaps compassion too, as we explore in this article.

Employee innovativeness is a complicated multilevel phenomenon and is commonly held to emerge from interactions between individuals and the work context (Hunter et al., 2007; Anderson et al., 2014; Mumtaz and Parahoo, 2020). Anderson et al. (2014) have investigated three broad categories of antecedent variables for innovativeness, namely, individual factors (e.g., personality traits, thinking styles, and motivation), task context (e.g., job complexity and job requirements), and social context (e.g., leadership styles and social networks). Earlier research has also offered a large number of variables as possible determinants of innovation. These factors are commonly divided into four broad categories: individual, job, team, and organizational levels. They all positively influence innovative behavior at different levels, sometimes independently but most often in interaction (Woodman et al., 1993; Anderson et al., 2004; Shalley and Gilson, 2004). Parzefall et al. (2008) have presented factors that influence employees’ innovativeness in the workplace, based on a literature search of 15 peer-reviewed journals (published between 2000 and 2005) and other relevant material, they have utilized the same four levels to classify the factors promoting innovativeness (see Table 1).

**Table 1:** Factors influencing employee innovativeness at work (Parzefall et al., 2008).

Broad categories	The factors positively influencing employee innovativeness
Individual-level factors	Ability (e.g., has certain cognitive capabilities, expertise, relevant task knowledge, necessary technical skills, and personality characteristics) and willingness
	Being open to new experiences, independence of judgement, a firm sense of self as creative, and self-confidence
	Being willing to try and accept the possibility of failing.
	Having internal force that pushes the individual to persevere in the face of challenges in creative work
Job-level factors	Autonomy
	Clarity of goals
	Lack of routine
	Suitably complex and demanding job
	Sufficient material resources and time
Team-level factors	Team and project-based work
	Deep-level diversity, i.e., diversity in skills and knowledge, or functional diversity is particularly desirable, interdisciplinary teams
	Team cohesiveness
	Good interpersonal relations and the quality of team member exchange relationships
	Trust
	Goal alignment between members
	Reflective orientation
O	Innovation strategy
	Organizational structure
	Organizational culture
	Team/organizational climate

Even though compassion is such a proven asset for teams and workplaces (reviewed in Section 2.2), here in the list by Parzefall et al. (2008), it cannot explicitly be found. Naturally, the organizational culture and climate, as well as team cohesiveness and trust, will certainly benefit from compassion, and thus further promote innovativeness. However, the need for more specific elaboration remains.

Turning from organizations more toward individuals we would ask, what really sets an innovative individual apart from others? What do they have more of? Carmeli and Spreitzer (2009, pp. 173–174) have noted three crucial conditions. First, when people have an opportunity to learn, grow, and develop at work, they are more likely to identify problems. They also want to solve them and develop new ideas. Learning, in terms of innovative working, means discovering new ways of working and being able to be creative. Second, energy and motivation are important elements in innovation. Energy strengthens the ability to transcend familiar roles and to think and act creatively. Promoting and developing new ideas thus requires energy, as innovation is a proactive search for new technologies, techniques, and processes. It is also often necessary to respond to criticisms and doubts about innovation (Dutton et al., 2001). Third, positive emotions broaden individuals’ repertoires of ideas and actions. Positive emotions broaden thinking, so they can alter cognitive functioning, which in turn affects action and behavior. Positive emotions can increase our intellectual, psychological, and social resources. The three aforementioned factors can play a key role in intrinsic human motivation, which in turn is highly relevant for innovative behavior. As Vinarski-Peretz and Carmeli (2011) state, motivation and commitment to innovative processes increase when people experience psychological safety and meaningfulness and are able to use their own physical, psychological, and emotional resources for the work to be done.

Not all organizations are the same. For instance, Bysted and Risom Jespersen (2014), studying public and private sector jobs in Denmark, Norway, and Sweden, found that in the private sector, innovation is driven primarily by career development, while, in the public sector, it is driven by meeting targets. Public sector employees perceive innovative work as risk-taking. Furthermore, the cultural environment matters, for example, specifically in Finland, innovation is often examined instrumentally, needing justification from an economic perspective (see Nieminen, 2013; Takalo, 2013).

It is, therefore, important that the atmosphere is sufficiently safe and empowering if we want to promote innovative work. Empowerment strengthens the autonomy of employees and the competencies needed for development work. Compassion plays a definite role here, for example, servant leadership fosters intrinsic motivation and a sense of autonomy in employees (Melwani et al., 2012; Paakkanen et al., 2020).

Indeed, in the explorations of innovation, compassion as a concept has to date received far too little attention. However, the phenomenon is not completely absent in the innovation literature. For example, in the context of workplace spirituality (Mitroff and Denton, 1999; Ashmos and Duchon, 2000; Milliman et al., 2003; Kolodinsky et al., 2008), a sense of meaning, belonging, and value-matching—which all require and are promoted by compassion—have been linked to innovative work behavior (Afsar and Rehman, 2015).

### 2.2. Compassion in innovation

Compassion is generally seen as a multidimensional phenomenon, involving cognitive elements (noticing or being aware of other people’s experiences), affective elements (feeling, sympathetic concerns, and empathy), and action elements (responding, readiness to help, acting to ease the suffering, and caring for others) (Kanov et al., 2004; Miller, 2007; Jazaieri et al., 2012; Dutton et al., 2014). There is no single definition of compassion; some notions, for instance, also put emphasis on the intermediate stage of intention to act (Jazaieri et al., 2012). At the core of compassion, there is a focus on the other and the wish for positive changes in their lives (Solomon, 1998; Miller, 2007). Avramchuk (2012) has studied compassion in the healthcare sector. The study found that compassion was constructed at both emotional and conscious levels. The general compassionate process was found to be as follows: a triggering event or circumstance is followed by an emotional experience and then a compassionate act. On the other hand, personal sources of meaningfulness were shown to reinforce the experience of compassion, and thus lead to more compassionate solutions to situations.

The effects and appearance of compassion in organizations have been researched in depth. Here, we look at the fruits of compassion that might come closer, particularly to creativity and innovativeness. Compassion is observed to have a positive effect on the processes of change and development (Smith et al., 2019), as well as in leadership development (Boyatzis et al., 2006). In leaders, compassion has been linked to leadership skills and capabilities, including servant leadership (Melwani et al., 2012). This is also proven in Finnish work culture (Paakkanen et al., 2020). Looking at the employees then, experiencing compassion from superiors has been proven to enhance work engagement, performance, and organizational citizenship behavior (Eldor, 2017). Furthermore, compassion has been shown to alleviate poor workplace climate and to strengthen decision-making capacity (Maitlis and Ozcelik, 2004), all needed in creating an environment for innovation. Compassion has also been found to have a positive effect on mutual caring in work communities, as well as on commitment to a work community (Dutton et al., 2006).

Innovativeness is neither just about individuals themselves nor is it simply about organizational structures. Echoing this, we regard compassion in organizations as processual and relational, as well as multidimensional. It is common to think of it as an individual characteristic, and a given individual as being either “compassionate” or “uncompassionate” (Kanov et al., 2004). However, the individual and organizational always mix, and for both levels, compassion is a more practicable talent than a trait. Indeed, individual- and team-level compassion has been shown to have an impact on the initiation of change processes, speeding up work processes, and developing organization-cultural compassion capital (Dutton et al., 2006; Lilius et al., 2011). Understanding the multilayered nature of compassion makes us better equipped to explore the synergy between compassion and innovation.

Compassion literally means “to suffer together.” However, some definitions of compassion, like Boyatzis et al. (2006), focus on encountering others, in various emotions. Thus existing definitions of compasson do not focus exclusively on negative emotions and responding to them. In the workplace too, participating in another person’s feelings and living alongside them entails the sharing of pain and stress, as well as joy and inspiration.

To stress this versatility and reflect the full scope of emotions involved in compassion—always needed in creativity and innovation too—this article incorporates in compassion the novel concept of copassion: the co-innovative, co-creative side of compassionate relationships (Pessi et al., 2022). Copassion refers to an affirmative response to the joy of another. It is rooted in the idea of shared humanity and intersubjectivity—but instead of focusing on sharing and alleviating pain and suffering, it focuses on advancing together, sharing success, enthusiasm, and inspiration (Kanov et al., 2004; Dutton et al., 2006; Miller, 2007; Jazaieri et al., 2012). Copassion, just like compassion too, also involves noticing, feeling, and acting, as well as sense-making (Pessi et al., 2022). This sister phenomenon of compassion is fundamental to innovation. For instance, it involves noticing the novel ideas and innovative potential a colleague might have, wanting to advance these together toward a shared goal, and carrying out the right actions, large and small, to realize this advance. With compassion, an organization is clearly more open to novel ideas, encourages innovation among all of its members, and is more prepared to use new ideas innovatively—in a word, more innovative. Copassion is not only an organizational trait but also a capability that can be strengthened, fostered, and utilized, just as compassion, mentioned earlier. Thus, in this article, we also examine the appearance of copassion when we focus on the connections between compassion and innovation.

As Avramchuk (2011) points out, compassion can be defined in different ways, and research should encompass its varied manifestations in the everyday life of a workplace. Furthermore, we state that in innovation, the copassionate side of coexistence. This concerns all of the three: the individual, the community, and the organization, is crucial.Thus, copassion is vital for innovation. For example, being able to expect your colleagues to share your enthusiasm about a new idea is crucial for summoning up the courage of presenting the idea to others.

Furthermore, the concept of innovation empathy gives us clues as to how compassion and innovation could be linked. In earlier research, as a theoretical construction, empathy has been observed to enhance innovation. The concept of innovation empathy refers to all forms of empathy that are related to how innovators perform in their work. Empathy can be cognitive, meaning the ability to put oneself in another person’s shoes. Empathy can also be emotional where it is about sharing feelings. These two components of empathy are intertwined (Montonen et al., 2014). According to Montonen et al. (2014, pp. 370–371), innovation empathy is above all the ability to take others’ perspectives into account. Innovation empathy helps to keep the customer’s or end user’s perspective in mind throughout the innovation process. On the other hand, it is important for innovation facilitators to demonstrate and maintain a climate of empathy, as it creates positive opportunities for the process and outcome: how innovators understand customers’ problems and seek to find solutions to them (Davis, 2006; Montonen et al., 2014). In stressing the importance of taking into account customers’ positive experience, studies of innovation empathy also underscore the relevance of paying attention to the synergy of copassion and compassion in exploring innovation.

Thus, this article focuses on innovation as a relational phenomenon involving a plethora of viewpoints (such as interpersonal relationships, shared aims, cultures, and processes) rather than approaching innovation as personal solitary achievements. Moreover, we approach this fascinating relational phenomenon by focusing on all of the aforementioned four levels: individual, job, team, and organization. In particular, our interest in this article (not yet in our data collection) then lies in understanding the role of compassion—including copassion—in innovation.

## 3. Materials and methods

Our aforementioned relational take on innovations constitutes the core reason for our data collection in a discussive setting within organizations. To collect the research material, nine focus group discussions on innovation were organized in Finland in 2017, gathering participants from a wide range of arenas: public sector, private sector, and third sector. To reach the public sector, five focus groups were organized in two smallish (*circa* 10,000–40,000 inhabitants) municipalities, three groups in one municipality and one in the other. They represented a variety of participants. In these public sector focus groups, the participants were not only public sector workers but also local small business owners or their employees, as well as town council members. Then, to reach the private sector, two focus groups were organized in a Finnish multinational pharmaceutical company. These focus groups consisted of both employees and the leaders of the organizations in mixed groups. Finally, to reach the third sector, we organized two focus groups with participants from various third sector organizations. These research sites were chosen among those who displayed interest in the study, and all organizations selected were large enough to accommodate focus groups. Focus group participants were recruited by the organizations internally with an open call.

Each focus group had 3–8 informants. Background information about the participants, such as age or gender, was not collected in the study. But in general, all participants were of working age, and there were both male and female participants in all focus groups. In addition to the groups, two thematic interviews were arranged with key persons of the same pharmaceutical company, as well as the aforementioned municipalities: one interview with two persons and the other with one person. All of the focus group interviews, as well as the individual discussions, were guided using a similar structure and cues (see below), and all lasted between 60 and 120 min. Altogether our research material thus consisted of eight focus groups and three thematic interviews. A total of 46 + 5 informants contributed to the material collection.

To examine our core phenomena as widely as possible and to keep it rooted in the everyday life of the organizations, we decided to use a cue containing elements of not only innovation but also the development of new ideas in general in the particular organization in question. The focus groups were presented with questions regarding innovative workplaces, in general, as well as innovation at their own daily work. This study uses responses to one of the questions: “What are the factors that prevent and the ones that promote the emergence and development of new ideas and innovation at this workplace.” The participants were first instructed to write down, on their own, the factors preventing and promoting, on colored sticky notes, using one color for the promoting and a different color for the preventing factors. After a few minutes of solitary thinking and writing, participants discussed their notes with each group. All participants took part in these non-structured, non-facilitated discussions.

The focus groups were voice-recorded (some also video-recorded) and transcribed into text documents. This material was analyzed using Atlas.ti qualitative analysis software and adapted the grounded theory methodology. Grounded theory is “a set of systematic inductive methods for conducting qualitative research aimed toward theory development.” (Charmaz, 2003).

The grounded theory involves recognizing and building categories (Dey, 2007), adding categories based on theory, and possibly also collecting new data during the course of research as a phase of “theoretical sampling” in the grounded theory methodology (Draucker et al., 2007). Grounded theory is especially well-suited for novel explorations of phenomena and their relations (Charmaz, 2006), such as this study.

The material was coded first by free coding: themes arising from the material. The material was divided into half, and each half was coded by one of the researchers, and then in the second phase, the codings were double-checked by the other researcher. After the first round of coding and during the whole coding process, the codes were grouped into larger thematic entities, such as ‘values’, ‘leadership’, ‘resources’, and ‘compassion’. Furthermore, more theoretically based coding—such as codes related to the different levels of innovation—was also added at a later stage. Then, the entire material was additionally coded for factors preventing and promoting innovation, with a purposefully broad scope. A key aim for all of our analyses was first to structure the mass of codes to find key themes in the material. The second key aim was to examine the co-occurrence of various codes at the textual, grounded level.

Two matters must be underlined: relating to our research aim, our data do not consist of facts but rather experiences and feelings; that is, what did the participants themselves consider preventing versus promoting factors? Furthermore, even if our interest lies in the particular role played by compassion and copassion, this issue was indeed not explicated in the guidance at all. Next, we present the results of our analysis, starting from the factors promoting innovation and advancing to the factors preventing innovation, and the emergence of new ideas.

## 4. Factors promoting innovation and the emergence of new ideas

### 4.1. Cultural practices at an innovative working community

In the material, the community and its characteristics seemed to be clearly the most influential factor in the emergence of new ideas and innovation. In the material, there were 180 quotations coded with “the work community,” 90 of them were also coded with “factors promoting innovation.” Other themes, such as “working culture,” “leadership,” or “values” generally reached 20–30 quotations connected to the promotion of innovations, so the difference is striking.

The core of an innovative working community seemed to be built around trust (19 quotations). Trust was in the data explicitly connected with openness, collaboration, communication, and sharing, thus promoting the emergence of new ideas from everyone’s point of view.

*By first being honest with oneself and the others, we build trust, and then we are capable of open and inspiring interaction. And then we can reach the goals which we believe in and are ready to work for them (9:7).* ^1^

Both community, more generally, and trust are fundamentals that clearly resonate with compassion—including copassion.

The sense of community and belonging was clearly elements of a good and innovative working community (nine quotations), but at the same time, the working community was seen “in action”; that is, people did not always write about a sense of community as such but more about its manifestation in everyday work. Such notions included, for instance, people being inspired together by each other (12 quotations) and by doing things together (19 quotations). As seen in the number of quotations and mentions, the actual deeds and manifestations of a good working community seem to be essential:

*More and more I’ve realized that it’s something we do together, and in a way we can get everyone involved, and everyone plays their own important role in it (7:84).*

The working community also promoted the emergence of new ideas in the form of a team, actually more in the form of a sense of a team, and as the support received from team members, as this informant states:

*It is also essentially linked to the kind of support you get from others, so that when you can be genuinely what you are, you also get support for yourself (5:99).*

Indeed, compassion is experienced to be pivotal here. The quote mentioned earlier also illustrates the key feature of a community: its relationship with the individuals it comprises. According to the respondents, on the one hand, an innovative working community needs the right individuals, but on the other hand, an open attitude to different personalities, even the more challenging ones. Some respondents also mentioned that strong personalities or powerful key figures can be either the strength and backbone of a community or a destructive force within it.

### 4.2. In between the individual and the community—Shared processes of innovation

In the material gathered, both in individual responses and the discussions, the interplay between individuals—with their own experiences and motives—and the whole community was the central framework for innovation, enthusiasm, and the emergence of new ideas. Compassion and copassion, in particular, are at play, as a base for the creation of a type of organizational atmosphere—something that is at times difficult to pinpoint precisely. The interplay is clearly explicated by the following respondent who works in the logistics department of a multinational company:

*(What promotes the emergence of new ideas is) first, of course, your own attitude and thoughts about wanting to change or develop things in general. – – Then, of course, the atmosphere, that you can suggest new things, and then that you get support from your supervisor or colleagues or whoever you are with, so that you can come up with ideas and try something new* (27:22)

On a larger scale, throughout the material, the interplay between compassion and copassion was evident, as well as the working culture, values, attitudes, atmosphere, meaningful work, and gains from work. This further validates our approach, which was to examine compassion as a holistic phenomenon: encompassing both compassion and copassion and reaching different levels of an organization.

With regard to the working culture, communication in many of its forms seemed to be essential for innovation and the emergence of new ideas. Good communication and transparency seem to foster inspiration and enthusiasm by creating concrete possibilities to view and develop further colleagues’ new ideas. Compassion specifically played a role here: are we able to encounter each other in a humane way? Even more so, innovations seemed to be linked to compassion, for example, the sharing of and reacting to positive emotions in drafting something novel together and in the sharing of wild ideas. For instance,

*it was transparent, making suggestions and initiatives. In the instant somebody typed an initiative on their computer, everybody saw it, the steps in the process, everything. You could see the comments instantly, was it accepted or not, what was the payoff. It fueled enthusiasm: ‘Oh, you could make an initiative related to this matter, we also have an idea, why did not I think of making an initiative before.’ The whole group got excited, and discussed the initiatives. So transparency is, for me, something. It’s good to see what others think, and from that new ideas are born (1:97).*

On the other hand, to listen and to be heard, the reciprocal dimension of communication, compassion, and mutual support was underlined in the material—both in the sense of encouraging every member to share their views and in the sense of the discussion as the key process in solving problems and in rethinking ways of acting.

*On the contrary, it’s probably a good thing that it has a little variation, someone comes along, someone from the outside who has not been there before and listens a little bit and says it out loud what the others do not, sort of wonder (2:185).*

How mistakes are handled was also central to creating innovation in the working culture, encouraging further the process of developing new ideas.

In connection with innovation, putting values into action, acting out, and fostering personal values, as well as living out values at the workplace, with colleagues were seen as key factors. Equally important was that work and its aims, and the organizational values would cohere with one’s personal values. For instance, respecting mutual helping needs to be shown every day. This was generally considered as affecting the motivation and commitment to innovate and create new ideas, and also the ability to identify with challenges in the field.

Of all the values inspected, openness was by far the most affluent with innovation and new ideas. It co-occurred with increased innovation 18 times in the material, whereas the next biggest value cluster, for example, gender equality was mentioned just twice in connection with increased innovation. An open atmosphere was seen to be essential at the workplace, to enable and facilitate discussion, to permit everyone to contribute, to permit healthy criticism, and to build trust and interplay in general. These two were even seen as the precursors to any innovative actions:

*By first being REAL to yourself and others >that’s how you achieve TRUST >that’s how you can have open and inspiring interactions >that’s how you achieve the goals you believe in and are willing to work towards (9:6).*

One of the leading arguments was: the more there are different persons, opinions, and views contributing to a situation, the more possibilities for new ideas there are. As this informant explains:

*then this openness, openness to appreciate different people indeed, it is also largely related to this kind of hierarchy. We value each link in the chain (1:21).*

Openness was also seen as a counterpart to strong hierarchies, which in turn were seen as preventing the emergence of new ideas. Openness was seen both as an intrinsic value as such and also as an aspect and quality of working culture in the everyday practices at the workplace. Furthermore, openness to sharing one’s worries as well as joys—the starting points of compassion and copassion—was vividly emphasized.

In general, attitudes were widely present in the material in connection with increased innovation, both as personal attitudes defining the individual’s way to work and shared attitudes in the community or workplace. The interplay between these two is evident. In particular, our informants underlined the role of a warm, encountering attitude toward colleagues, both compassionate and copassionate ones. Indeed, the two strongest, most visible, and also most widespread attitude clusters in the material, in general, were: (1) positivity in general and a positive attitude toward other people and (2) challenging/questioning authority and prevailing norms/practices. Interestingly, in proportion, questioning authority (4 out of 15 quotations) did not co-occur as strongly with innovation as did positivity (17 out of 31 quotations).

The role of positive attitudes in compassion was apparent. For instance, positivity in relation to others and the community—individuals affecting others—was emphasized:

*A person who is positive and fair and, like, happy – one who has positive energy – will surely get the others to work their best, too (5:155).*

Positivity in relation to the quality of work and innovation was also noted as an eminent promoter of innovation:

*A positive attitude to work has an effect - -even if the work is difficult and challenging, people want to work with it, not run away or push it aside. People have motivations and enthusiasm to solve problems (1:82).*

However, saying ‘yes’ to everything was not a key to innovation, according to our material. If conducted with respect and in a compassionate, humane spirit having the attitude to challenge or question each other can also play an essential role in the fostering of innovation. Other attitudes contributing to innovation were: a willingness to make one’s best effort, seeing the importance of equal treatment for everyone, and justice in the workplace in general. These too powerfully resonate with compassion: valuing each human for who they are; being open to other people’s experiences; helping others; and taking others into account.

*And listening in general, you do not just listen, you really listen, you are present, so you stop and you really know that I’m listening to what you are saying, so that’s a pretty big thing in my opinion.*

In general, the atmosphere was experienced to have a huge impact on innovation (10 + 16 quotations), according to our informants, for example, making it possible for all members of an organization to express their ideas and encourage design-thinking. This demands psychological safety and trust in the compassionate approach from colleagues: Do I trust that this is a safe, humane place to share even wilder ideas? Interestingly, our material acknowledged that everyone can affect the atmosphere, and then the atmosphere in turn has an effect on all the members of the community. It was considered that compassion and copassion in everyday life are the responsibilities of each and every member of an organization. This view was explicated, for instance, by one of the corporate leaders participating in the interviews:

*The day has to go with a good mood. I sometimes thought that I was the creator of the mood but the people are the creators of the mood, that I have nothing else to do but maybe show a little example of what the mood is but that the people then create it themselves (7:73).*

The sense of one’s own work belonging to a bigger entity and the notion that the work has an effect on something important in life were evident in the analysis in connection with innovation and grouped as finding meaning in work. Experiencing meaning in work was explicitly connected with motivation:

*but perhaps the single most important thing that encourages or possibly discourages innovation, is motivation. Somehow it seems that, in addition to the salary, the purpose of the work you do, − from your own point of view and hopefully also from the point of view of the organization, and in the best case also from the point of view of the customer – is to produce something that you yourself feel is meaningful at some point in the chain. Preferably at all points. If there is one factor missing, the motivation can be quite weak. The salary is probably not motivating after the basic needs are met (27:29).*

Innovation and the emergence of new ideas were also connected with how and in what ways daily working life is rewarding. With regard to the crucial benefits of work, the respondents mention personal growth, the satisfaction of inspiring others and helping them learn and grow, and the sheer enjoyment of the work.

*If I were to put it this way, I’ve also experienced that there’s a certain growth here. Spiritual growth too. Depending on what you have been through at what stage you have been through, the things that might be objects of joy and things that are satisfying, they come in a different way.*

Our analysis suggests that what people perceive as the benefits of their work is largely connected to the impact of work: noticing the impact and seeing the possibility to have an effect *via* work, appreciating it, and sharing the goals and values of the work. This, in turn, would increase the motivation to make changes in the workplace and to benefit from the work even more.

### 4.3. New ideas as a result of—and promoted by—Organizational qualities: Structures, resources, and bureaucracy

Promoting the emergence of new ideas was also seen as being a result of certain organizational features, such as structures, leadership, goals, and resource management.

When speaking of the structures and organizations that support the emergence of new ideas, the informants used the term ‘flexible organizations’, referring to a workplace open to change and new innovations where it is easy to cross boundaries and bring forward one’s own ideas. Also seen as important were how ideas and initiatives were handled, and whether the organization had any standard protocol to advance new initiatives and feedback.

*As promoters, so the leadership has a big role, how to give space for new ideas and give the opportunity to discuss in general. And the functionality of the whole organization: how you handle and act upon initiatives, for example (1:77).*

Our material highlights the importance of humane and compassionate organizational structures for innovation to emerge.

The importance of having goals and visions in an organization, and explicating these in the workplace, was one structural feature that was quite pronounced in the material in connection with innovation. Equally important were the goals in the organization that the leader advances logically toward them and is open about them. Furthermore, sharing a goal with colleagues and the organization, in general, was connected to the emergence of new ideas—mainly through motivation to develop further the organization and to see the results.

Leadership was experienced as a part of the structures of an organization, and at the same time, a question of individual behavior manifested, e.g., in leadership skills. The styles and qualities of leadership promoting the emergence of new ideas were distilled into five elements, in particular, leadership that is encouraging, stimulating, honest, rewarding the employees, and equality toward the employees.

*Mutual trust is really important. Then when you give feedback, it must always be honest, is it criticism or praise, so always if you are honest so it becomes genuine. Then in fact, managers must feed the strengths of all their staff, they must always pay attention to what someone is doing badly or otherwise, try to guide it in the direction that it uses their own strengths, so you get really good results. Rewarding is a good way to take things forward, but then, uh, for the supervisor so equal treatment for all employees, so everyone is equal, you can not give someone better feedback, something extra for some things that they are a factor of equality, so it is something like improving the working atmosphere, and then all that competence update training and other factors that promote competence, and then work, a good working atmosphere which others have mentioned (4:130).*

Leadership ideas such as a compassionate and servant leadership style were clearly seen in the material as promoting innovation.

The single biggest structural feature and resource that promote innovative behavior and the emergence of new ideas in the material were time-dedicated to innovation. The emergence of new ideas was linked to tranquil moments and ‘idle’ time or time to pause, giving space not only to individual thought processes but also to bounce ideas around with others.

*They always say that you have to have time for it, you have to take time to rest your brain a bit. That’s probably why we have a lot of them [ideas] in our free time, because then it’s just time. You have to be relaxed enough to be innovative. You stop to think properly about a process. How can you do this now? What do we do about this? Now put everything together. Let us get this right so we do not have to complain about it anymore (27:98).*

Also very important are informal, regular get-togethers such as coffee breaks and other circumstances to meet colleagues without a strict agenda—in contrast with organized team building events or recreational days, which take place with longer intervals.

*Quite a few of our projects have started from the coffee breaks: Somebody weighs in [with]an idea, and then someone else gets inspired, someone writes it down as a report, and encourages others to check this and that, And soon we have a project going on (2:116).*

Our material showed that using time for innovation created possibilities for both compassion and copassion. ‘Idle’ time not only promoted compassion between team members but also made compassionately visible the humane needs and capabilities of the individuals. For encouraging innovation, the structures regarding time use should be designed with compassion in mind.

## 5. Factors preventing innovation and the emergence of new ideas

### 5.1. Cultural practices as barriers to innovation

The barriers to new ideas and innovation that emerged from the data were habits and routines of individuals and communities – habits that were perhaps too strictly adhered to. People are used to working in certain ways, even if the way is no longer effective and efficient. There was not always a willingness to change and reform ways of working and behavior. Some interviewees spoke of resistance to change.

*It’s not a very fruitful basis for getting something new and inspiring, so usually people will go back to the solutions that have been tried and tested, and go forward with those, because at least they have been reasonably successful with them in the past.*

Resistance to change, or reluctance to change in general, was often thought to be due to a lack of understanding of the needs and implications of change. There was too little communication about the need for change and development, and people were not involved in the development process. Ideas often came from the top down, with the management generating ideas and directions for development, without other levels of the organization being able to influence and be involved at the point of inception.

Some sectors were described as having traditional, work-related, and cultural patterns of working in certain ways. These were professional traditions that were respected and not to be abandoned:

*If we are talking about something like a municipal organization, like a care institution. When young people come there, and there are these experienced people involved, so there you are a prisoner of this kind of group, a group with fixed habits. After three days you stop trying to introduce any reforms there. You’re crushed and the old ways continue, and the action is still the same. And the young are not happy (3:12).*

The interviews also revealed that working in an individual-oriented way or sticking strictly to the boundaries of one’s own job description was also an obstacle to innovation. Interviewees described situations where people were not prepared to move beyond their own tasks or to broaden their view of what their own tasks entailed. The boundaries were strictly adhered to.

*Individualism is a kind of slowing factor. If everybody is just strictly in their own box, if they take care of just their own domain, it is indeed a slowing factor. We should get the teamwork going on (2:81).*

There were a significant number of mentions in the interviews of how the atmosphere and attitudes in the workplace affect innovation. An atmosphere that is stimulating and conducive to new ideas will be undermined by cynicism, ill will, jealousy, and mistrust among people. Some reported experiences of competition within the workplace. A suitable competitive spirit can foster renewal, but competition can also set limits on what people are willing to share, such as their own skills.

### 5.2. In between the individual and the community—Barriers to shared processes of innovation

Many interviewees pointed out that innovation is undermined by shyness and fear of bringing one’s own ideas to the workplace. Many interviewees felt timid about how their ideas are received in work communities. Some interviewees were afraid of being rejected and others of being ridiculed. Some interviewees felt that there was a fear of failure and of making mistakes in the workplace. It was precisely the risk of making mistakes that were associated with new things, as this informant states:

A f*ear of failure and, for example, if you are really stuck in a certain pattern, perhaps because of time constraints, you just stick to certain routines. And then if you spend so much time on routines that you do not even have time to think about anything else, then that of course hampers innovation (27:20).*

Concern, timidity, and fear of brainstorming were identified, as being due to mistrust, a negative atmosphere, and poor team spirit in the workplace. On the other hand, timidity, for example, was perceived as an individual characteristic. Some felt more shy, quiet, and introverted in large groups. The acceptance, support, and encouragement of the group had a significant impact on the ability to express their own thinking.

Some interviewees pointed out that sometimes there are very strong personalities in working groups and teams who take a strong position, use their own voice and dominate common situations and discussions. These strong personalities could be drivers of new ideas, but they could also undermine the courage of others to share their own ideas or otherwise dampen the views of others:

*if there is a lot of brainstorming in the group, if there is a kind of imbalance – if there is someone who is too loud or dominant – it may silence the others in the group (28:17).*

Some interviewees mentioned that there are always individuals in work communities who “shoot down” anything related to innovation and change. Individuals could be a major obstacle and blocker to moving ideas forward. At times, personal disputes and grudges could also prevent ideas from moving forward. One example was given from the municipal policy side:

*In a small municipality, sometimes even the cottage plot issues can bother some decision-makers. If some of them did not get permit for their own cottage, they will say no to the permits of others, no matter what (2:133).*

The dynamics of working groups and teams were considered to be influenced by the motivation and resources of the individual. Shared innovation was not promoted if the person was not motivated or otherwise not invested in community situations:

*Just using devices like mobile phones has an impact on the whole atmosphere. If the intention is to come together to innovate something, then you would have to try to find a side of yourself that wants to contribute to it. (27:14).*

In other cases, some people just could not cope with or be tired of their own work. A lack of resources was seen as a serious obstacle to creating and developing something new. Several interviewees highlighted the importance of personal wellbeing in innovation:

*A barrier can be that if you are somehow bored with the job, you are tired of the work, then you do not have enough resources to think about it in a new way so that you just perform it in the way it is, because the easiest route is to go the same way that has always been done before (4:27).*

### 5.3. Preventive organizational qualities: Structures, resources, and bureaucracy

Most of all, the people involved in the group interviews talked about how different legislation, norms, and guidelines in organizations and society hinder the development of new ideas. Many talked about bureaucracy as being a barrier to innovation. Bureaucracy was something many had encountered in both the public and private sectors. Our study included a private sector organization whose sector was particularly heavily regulated.

*If we go by all the standards, there will be no innovation, and no new thinking will be allowed under any circumstances.*

*So it is perhaps just the general operating environment, laws and rules that prevail in the pharmaceutical industry that sometimes limit the scope for good ideas and thoughts, but if you cannot do it, you cannot do it (28:16).*

On the other hand, it was not only the legislation and norms that were seen as problematic but also the strict interpretation of the law in its implementation. Finnish officials were found to have a special obligation to implement the instructions to the letter:

*So there will be stricter regulation when the law itself is and then when it is, there is this stricter interpretation, so it happens at some stage that when the law is reformed, the stricter interpretation will be in the new law, that the legislature is originally lighter in this law when enacting, but then when implemented, so we Finns enforce it even more strictly (2:54).*

Strict laws, regulations, guidelines, and standards created a very narrow space in which to operate, to create something new, and to do things differently, even if it was found that previous solutions did not work. Bureaucracy was also perceived as a barrier to taking new ideas forward. Bureaucracy introduced a culture of caution and timidity in trying new things. People acted as if their hands were “tied” to fulfill their official duties and not to break the regulations.

*But that causes a very cautious spirit in this kind of municipal activity.--- Because then again, if you do something different, someone may complain about it. If the complaint goes through and shows that you treated community members unequally, you lose. And that creates a very cautious approach to anything new and anything innovative (2:60).*

In all of the group interviews, there were also some aspects of the organizational structure that undermined the possibility of innovation. The multilayered nature of organizations, hierarchical structures, and the distance of decision-making from everyday work and practices were perceived as problematic. In terms of reform and change, decisions were often taken by people who were not in touch with the practical issues and challenges.

Another key area that emerged as a barrier to innovation was that of resources and in particular the lack of resources of various kinds. The biggest challenge for many people was time. Many people felt that they were so busy that they did not have the time and resources to develop something new. On the other hand, time pressure created a situation where there was no more “idle” time—in this article referring to non-structured and perhaps seemingly non-productive time—to be creative and innovative. The generation of new ideas was seen as requiring free spaces and moments to think, brainstorm with others, and bring together different ideas and thoughts. Many people spoke for change. There used to be more time for sharing in work communities or networks, but now there is too little time for joint brainstorming.

*Then I feel hurried. I feel like there’s so much to do that I cannot think of everything until the end, if there’s a project. What do I want to do. I just do not have time. It’s disgusting. Then the processes in here. They limit quite a lot of what you can and cannot do (27:12)*

1. Another major lack of resources that several informants referred to was money. Without financial resources, many new ideas cannot be implemented. The lack of financial resources did not foster innovation. Access to information, skills, and training was also identified as resource gap. Access to information was linked, *inter alia*, to difficulties in communicating and interacting with people.
2. *There is not enough planning skill, there is not enough skills to control processes. We’re at the point that we run out of skills. Perhaps a pilot was made, but how to proceed? That’s the difficult thing. (2:74)*

Some interviewees said that it is a waste of resources not to evaluate and reflect on the work done, and the project or to reflect on, for example, what has been learned and in which areas further development is needed. The way the resources available are used is also an issue. Too little thought is given in work communities and organizations to whether resources are being used in line with objectives efficiently and effectively.

## 6. Key factors of innovation and their relations to compassion

Above, we have examined the factors promoting and preventing innovation in the organizations we studied. These are summarized in Table 2 below, with promotive factors on the left-hand side and preventive factors on the right-hand side. However, as noted earlier, our informants also revealed the factors that they experienced as playing a part in both preventing and promoting innovations. Thus, the middle column of the table contains the common denominators for promotive and preventive factors.

**Table 2:** Promotive and preventive factors for innovation and their common denominators.

Promotive factors	Common denominators	Preventive factors
-Explicated & shared goals, matching values and action -Just leadership	(A) Structures, strategies & leadership	-Not enough shared goals and actions aligned with them -Rigid structures in organization
“idle” time, time dedicated for innovation and reflection, time for sharing with team members	(B) Resources, especially time	No time to think, no “idle” time
-Positivity, copassion -Teamwork, support -Openness	(C) Working culture: atmosphere, attitudes, competition	-Negative attitudes -Individualism, not enough support and encouragement -Unhealthy competition
-Reciprocal and informative communication -Experiencing meaning and benefits of work -Sufficient prosocial skills and behavior of individuals, taking individual differences into account	(D) Dynamics of interaction between individuals and in the community	-Limited communication, fear to express ideas, -Lack of motivation, not experiencing meaning at work -Not being well at work, insufficient personal skills or resources

The core findings of this article are fourfold. First, as shown in Table 2, we found that the factors promoting and preventing innovation have a lot to do with each other. Many of the factors that promote and hinder innovation are positive and negative aspects of the same phenomenon—either the existence or non-existence of it. The strongest drivers and barriers to innovation occur simultaneously in the areas of individual–community relations, communication, and organizational climate and culture. In addition to these core factors, some clear ‘fringe factors’ emerged. These would either need to be eliminated for innovation to flourish (preventive factors) or would be an important booster to be implemented (promotive factors).

Our second key finding is that the common denominators of promotive and preventive factors were: (1) the strategy and structures within the organization, (2) resources, especially time, (3) working culture, and (4) the dynamics of interaction between individuals and the community. These four factors were found to be the key or core factors, which are capable of either boosting or preventing innovation, and at the same time being deeply connected with compassion. Many of these factors are present in all types of organizations, and thus, they are pivotal for either creating or hindering novel innovations.

Our third key finding is that the four factors are interlinked. As some of our informants explicitly noted, for example, openness in the interaction between team members might lead to an improved working culture, which might lead to better resource use and even to structural transformations in the organization. In our analysis, several different patterns of connection between the factors were found, not only linear or causal connections. This interconnectivity is characterized by intertwinement rather than a clear causal process. Thus, for promoting innovation in organizations, it is not enough to focus, for example, on strategy alone or merely to develop the working culture—all of the factors must be taken into account.

Our fourth and final key finding is that compassion is a vital feature of promoting innovation within those four recognized aspects. The results of our research show that innovation is linked to compassion in different ways. Compassion is strongly expressed not only as an individual’s internal experience but also as a relationship between individuals and communities and as a community experience. Miller et al. (2012) point out that because compassion is other-oriented in nature and is very much a human emotional experience, compassion also acts as a prosocial motivator that encourages the search for solutions to problems. Prosociality means, among other things, that people are willing to take into account information that others possess. It can also increase an understanding of information from the perspective of others and identify differently with the situations one wants to solve and help. Expanded perspectives can increase cognitive flexibility, willingness to take risks, and openness to the complexity of different situations (De Dreu et al., 2000; Grant and Berry, 2011; Polman and Emich, 2011; Miller et al., 2012).

Compassion can facilitate the integration of new and different ideas or creative approaches to problems. Openness to different ideas allows for integrated thinking about solutions (Miller et al., 2012). In the factor of strategy and structures, both the design processes and their end results should be compassionate to create possibilities for innovation. For innovation to emerge, the design processes should be inclusive and informative, and the organizational structures should be one’s promoting compassion and connections. In the factor of resources, the allocation should be fair and should encourage their use for compassion both toward oneself and others. Moreover, in the factors of working culture and interaction, compassion will need to be a core element of everyday life and organizational development, present both in practices and values for innovation to emerge.

## 7. Conclusion and discussion: Compassion as a key to innovation

Our analysis shows that innovation is profoundly and diversely connected to compassion. Particularly copassion – an element of compassion – is connected to innovation. Particularly copassion is seen by the informants as a key feature in balanced, healthy workplaces. This is in line with previous research. Earlier studies, including the field of organizational research, have found that, at a collective level, compassion is the ability and strength of individuals and organizations to respond to changing situations, in times of uncertainty, for instance, how to direct different resources, particularly during difficult times (Dutton et al., 2006). Compassion has been found to activate an organization’s capacity for appropriate flexibility in changing situations (Powley, 2009) and to contribute to organizational performance improvement and learning (Dutton et al., 2006; Powley and Piderit, 2008).

Our core contribution lies in revealing and highlighting the multidimensionality of compassion regarding innovation. First, both compassion and copassion—that is, reacting to both the suffering and joy of others—are pivotal factors for promoting innovation. Second, we found that compassion is deeply interwoven at all levels of organizations: in the dynamics of interaction, in the working culture, resource management, and organizational structures, etc. It can be advanced at all or any organizational level, and previous research has shown, prevented, and conversely destroyed in any of them (see, e.g., Singh et al., 2018). Compassion is neither a single variable or practice that could be added to an organization nor should it be thought of only after ‘the productive work’ or ‘the core tasks’ has been taken care of. For innovations to flourish, compassion is to be cultivated throughout an organization. Third, compassion is in many ways in a key position regarding innovation: the existence of it promotes innovation, but the lack of it powerfully prevents innovation. Compassion, thus, is not only a ‘booster’ or something extra but also a key element of a functional organization.

These findings have clear implications for the study of compassion in organizations, encouraging focus on the interplay between different levels of organizations. Innovations are not the responsibility of any single role or department, naturally. Furthermore, our findings have implications for organizational development and practice. There are strong reasons to take compassion seriously as a key asset in organizations, not as an expense, but rather in relation to flourishing creativity and innovation.

## 8. Limitations of the study

Our study has some significant limitations, particularly related to the research material. Although our material was collected from various types of organizations in the business and public sectors, we mainly focused on expert tasks and collected material in one specific cultural and geographical context. The theme should thus be studied further in different cultural contexts, especially those beyond ‘white, educated, industrialized, rich, and democratic’ (WEIRD) settings. However, in our exploration of organizations in various sectors, we interestingly found no significant differences in the experiences of relating innovation with compassion. This indicates that with compassion, we are indeed looking at a basic human need and capability, which is equally relevant in all workplaces and organizations.

## Data availbility statement

The raw data supporting the conclusions of this article will be made available by the authors, without undue reservation.

## Ethics statement

Ethical review and approval was not required for the study on human participants in accordance with the local legislation and institutional requirements. The patients/participants provided their written informed consent to participate in this study.

## Author contributions

JS and EJ were largely responsible for the data collection and analysis. All four authors took part in writing this article and agreed to be accountable for the content of the article.

## Funding

This study was funded by the Finnish Funding Agency for Technology and Innovation (2684/31/2014).

## Conflict of interest

The authors declare that the research was conducted in the absence of any commercial or financial relationships that could be construed as a potential conflict of interest.

## Publisher’s note

All claims expressed in this article are solely those of the authors and do not necessarily represent those of their affiliated organizations, or those of the publisher, the editors and the reviewers. Any product that may be evaluated in this article, or claim that may be made by its manufacturer, is not guaranteed or endorsed by the publisher.

## Footnotes

^1^The numbers refer to coded text segments, ‘quotations’, and are kept here because of traceability to the raw material.
